# Surgical treatment of patients with chronic rupture of the pectoralis major muscle tendon. Prospective comparative study with 2 years of follow-up

**DOI:** 10.1093/jscr/rjae093

**Published:** 2024-03-16

**Authors:** Alberto de Castro Pochini, Benno Ejnisman, Carlos V Andreoli, Andre F Yamada, Ivan R B Godoy, Moises Cohen, Maria T Seixas, Paulo S Belangero, Debora C Hipolide

**Affiliations:** Orthopaedic Department, Federal University of Sao Paulo, Brazil; Orthopaedic Department, Federal University of Sao Paulo, Brazil; Orthopaedic Department, Federal University of Sao Paulo, Brazil; Orthopaedic Department, Federal University of Sao Paulo, Brazil; Orthopaedic Department, Federal University of Sao Paulo, Brazil; Orthopaedic Department, Federal University of Sao Paulo, Brazil; Pathology Department, Federal University of Sao Paulo, Brazil; Orthopaedic Department, Federal University of Sao Paulo, Brazil; Psychobiology Department, Federal University of Sao Paulo, Brazil

**Keywords:** comparative study, muscle strength, pectoralis muscles, rupture

## Abstract

To compare outcomes between autologous fascia lata and autologous hamstring grafts for chronic pectoralis major muscle (PMM) rupture repair, and perform histological, and imaging analyses. Forty male patients with chronic PMM ruptures (time since injury ranging from >3 months to 5 years) and a mean age of 37.3 years (SD = 9.7 years) were evaluated. One group (20 patients) received an autologous semitendinosus graft, and another group (20 patients) received an autologous fascia lata graft for PMM reconstruction. These patients with fascia lata grafts by Bak ^2^criterium 60% of the patients presented excellent results, 20% presented good results, 15% presented fair results, and 5% presented poor results. In the hamstring group 65% of the patients presented excellent results, 30% presented good results, and 5% presented fair results. In this comparative study, no difference was observed regarding the functional result, image, and histology between groups.

## Introduction

In the last years, the diagnosis of pectoralis major muscle (PMM) ruptures is increasing. It is associated with a large number of fitness centres, CrossFit, and strength sports [1–9].

The appropriate early diagnosis involves an appropriated physical examination. The bruises usually occur in the arm region, extending from the lateral aspect of the chest for 4 cm, and can reach a large area of the arm and forearm [4–11].

The most useful image tests are magnetic resonance imaging (MRI) and ultrasound. However, the correct and accurate diagnosis needs of radiologist doctor analysis with a high level of experience in musculoskeletal injuries [12–19].

The current literature describes good results for the surgical treatment of both acute and chronic injuries [19–25].

**Figure 1:**
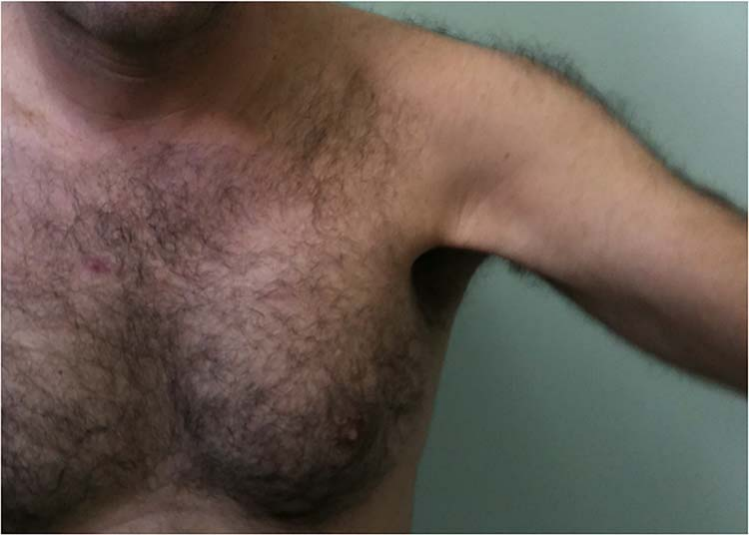
Patient practicing jiu jitsu with chronic rupture of the pectoralis major muscle tendon.

**Figure 2:**
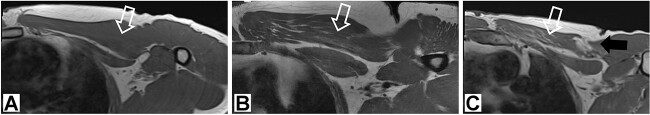
1.5 T T1 magnetic resonance image showing chronic aspects within the pectoralis major muscle.

**Figure 3:**
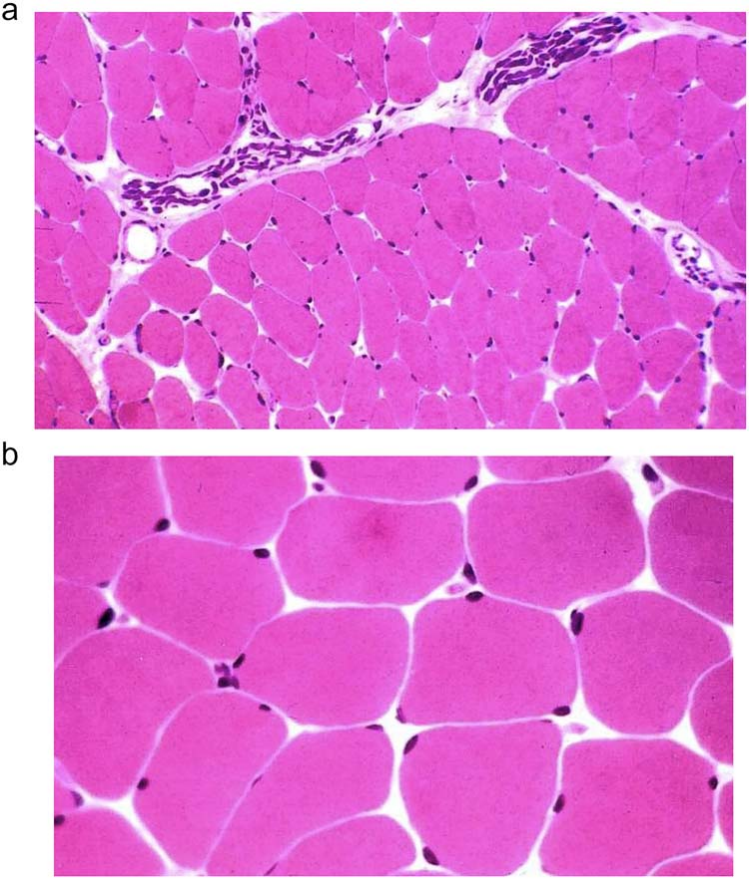
(a and b) Optical microscopy image showing haematoxylin eosin stained muscle from chronic cases of the pectoralis major muscle.

In this study, we presented a prospective comparative study of 40 patients underwent anatomical reconstruction of the PMM tendon using autologous fascia lata graft or autologous hamstring graft [15, 16, 22]. All patients have presented chronically ruptured muscles (Fig. 1) and were evaluated with MRI (Fig. 2*)* before the surgery. All patients underwent histological evaluation of the pectoralis tendon in a biopsy took during the repair surgery (Fig. 3*)*.

## Case series

This comparative prospective study was conducted between 2015 and 2021 using a STROBE checklist. Forty men with delayed chronic PMM rupture (time since injury ranging from >3 months to 5 years) and a mean age of 37.3 years (SD = 9.7 years) were evaluated. All lesions had occurred in the bench press exercise or CrossFit training.

The inclusion criteria were total or near total chronic rupture, visualized by an MRI, in weight training or CrossFit practitioners aged 23–45 years.

The exclusion criteria were chronic injuries with a retraction higher than the nipple line on an MRI in the T2 axial plane and failure to follow-up at 6 months post-operatively. Patients who did not return for follow-up and examination were removed from the study.

One group (20 patients) received autologous semitendinosus grafts whilst another 20 patients received autologous fascia Lata grafts for PMM reconstruction. The allocation criterium to each surgical group was convenience, according with the availability of instruments, implants, and equipment in the hospital. The difference between both surgical techniques and the difference between the grafts were explained to all patients. The autografts have been chosen and greafted from the thigh (fascia lata) or leg (hamstring).

Of the 40 patients in the study, 20 underwent histological evaluation to evaluate degenerative changes in chronic muscle injury. These samples have been compared with 10 control patients who were not weightlifters.

The surgeries were conducted by same surgeon experienced in this type of injury. This study was approved by and registered under CAAE number X. The post-operative follow-up was a mean of 30 months (24–60 months) [5].

## Surgical technique

### Fascia LATA

**Figure 4:**
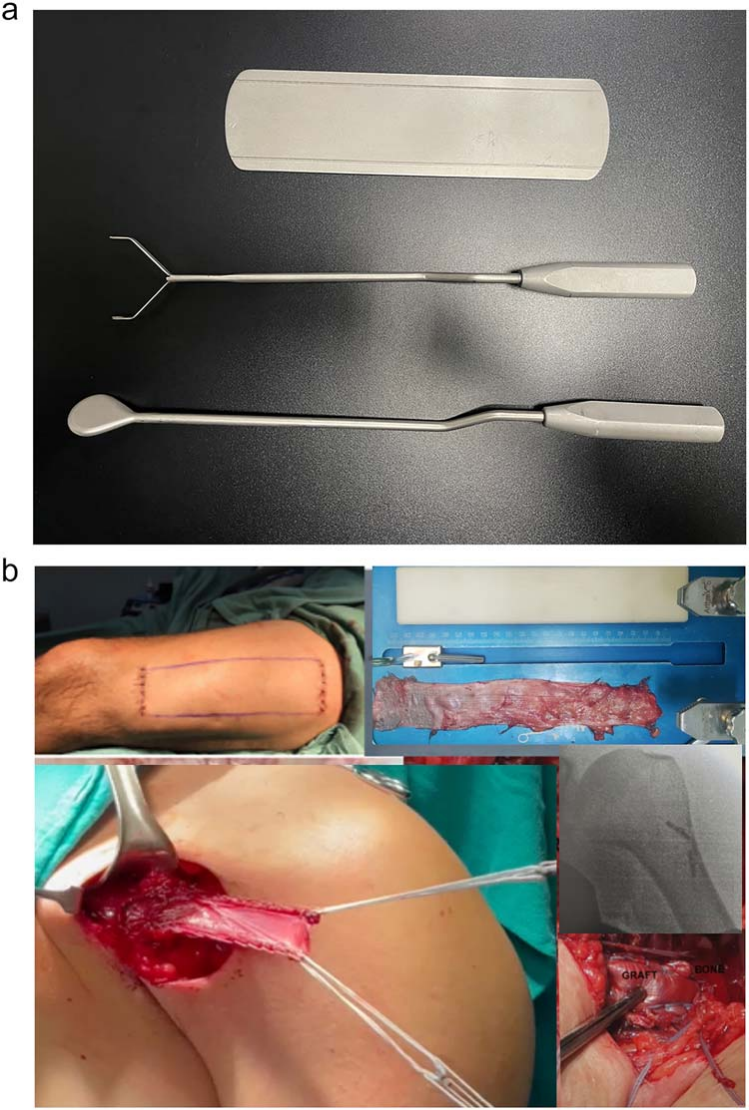
Images of the surgical technique for extracting the fascia lata with an extraction device Larson ® (a) and reconstruction of the pectoralis major muscle tendon using a cortical button and autologous fascia lata graft (b).

In patients underwent autologous fascia lata graft excision [1], two 5 cm lateral and transverse incisions were made in the thigh. The first incision was 5 cm and the second 25 cm proximal to the knee joint line. For this kind of reconstruction the graft must be 20 cm length. The fascia lata graft extraction followed the procedure described by Valerie Su-Lin Tay [22] with a small modification. We used an extractor tool developed by our group specifically for this purpose (Larsson®) (Fig. 4a). For PMM reconstruction, an axillary incision was made in the injured shoulder region. The muscle stump was sutured with an anchored non-absorbable #2 FiberWire® (Arthrex) Krackow suture, passing through the fascia lata graft, and folded three times in the proximal and distal regions of the graft edge. We reinsert the PMM with the fascia lata graft using 3 Pec Buttons® (Arthrex) and #2 FiberWire® threads, followed by closure by planes. All patients were kept with aspiration drain in the post-operative period for 24 h.

### Knee flexor technique (semitendinosus/GRACILIS)

The semitendinosus and gracilis tendons were grafted with the conventional technique [18] . The reconstruction was performed

by axillary approach. The semitendinosus and gracilis tendon graft were passed through the muscle, ~3 cm from the lateral border of the torn muscle [18]. The graft could then be carefully passed through the tunnels, always perpendicular to the humerus. It prevents the exit from being too proximal to the distal cortex and to the skin. After the proximal and distal wires emerge, the pectoralis major muscle is tensioned and adjusted.

All patients were kept with aspiration drain in the post-operative period for 24 h.

### Rehabilitation

A regular sling was recommended for 6 weeks, and passive physical therapy was performed for up to 8 weeks. After this period, active assisted movement was initiated. Weight bearing was allowed at the limit of pain for the ambulation with or without crutches.

### Functional outcomes and isokinetic test

The patients were evaluated in terms of functional outcomes according to Bak’s criteria, which include functional and aesthetic aspects and an isokinetic test [2]. The isokinetic evaluation was performed in a Cybex dynamometer at 60 deg/s (model 6000, Lumex Inc, Ronkonkoma, New York). Patients in the surgical group were subjected to these tests 6 months post-operatively. Patients in non-surgical group had this test 6 months after injury.

We use the Bak criteria for functional assessment, as well as several other studies [2, 4, 7, 8]. The criteria observed were: Excellent: no symptoms, normal range of motion, no cosmetic modifications, no adduction weakness, able to return to sports activities. Good: subnormal range of motion, no cosmetic modifications, 20% isokinetic peak of torque deficit. Fair: limited range of motion, not able to return to sports activities, poor cosmetic state. Bad: continuous pain, in need of surgery revision.

### Magnetic resonance image of the affected muscle

We performed MRI image analysis in 20 patients underwent surgical treatment. Those patients were randomized from all surgical patients in a ration of 2:1 with 10 non-weightlifting athletes’ controls. We used MRI to assess muscle degeneration prior to surgery using a protocol designed for PMM evaluations. For this randomization we used randomizer.org in a simple way. All patients evaluated with MRI had histological evaluation also. All MRI were performed using the same 1.5 T MRI machine (Siemens, Erlangen, Germany); the parameters are provided in Table 1 and degeneration evaluation in Table 2.

**Table 1:** Magnetic resonance parameters.

Pulse Sequence	TR (ms)	TE (ms)	NEX	Matrix	Thickness (mm)	FOV (cm)	Bandwidth (Hz)	Echo train
Coronal FSE T2FS	3000	49	1	512 × 256	4	22	250	6
Axial FSE T1	600	11	1	512 × 256	4	26	296	3
Axial FSE T2FS	3000	49	1	512 × 256	4	26	250	6
Sagittal FSE T2FS	3600	52	1	512 × 256	4	22	250	6
Coronal T1	500	15	1	512 × 256	4	30	122	1

TR: repetition time, TE: echo time, NEX: number of excitations, FOV: field of view, FSE: fast spin echo, T2FS: T2-weighted fat-suppressed sequence.

**Table 2:** Histological classification used in this study.

Muscle degeneration	Classification
1 = Presence of atrophic fibres	0 = Absent
	1 = Mild <25%
	2 = Moderate 26–50%
	3 = Severe >50%
2 = Ischemic changes	0 = Absent
	1 = Present
3 = Fatty infiltration	0 = Absent
	1 = Present

### Histopathology of muscle fragments

We performed histological analysis in muscle fragments from 20 patients. Those were fixed in buffered formalin, embedded in paraffin, and stained with haematoxylin and eosin (H&E) (Table 2). These were the same underwent a MRI to study muscle degeneration. For the histological evaluations of muscle degeneration, two samples from the retracted muscle stump were collected from 20 patients at the time of surgery; five samples were damaged during slide preparation, leaving 15 patients for histological analysis of muscle degeneration.

## Statistical analysis of magnetic resonance images, histological analysis, and controls

The groups that received a autograft fascia lata graft and knee flexors were comparatively analyzed by the first quartile, the third quartile, the student’s *t*-test, Fisher’s exact test and the non-parametric Mann–Whitney test.

The mean age at the time of injury was 32.9 [6, 7] years old (22–44) (Table 3). In this series, 33 (82.5%) were injured during bench press exercises and 7 (17.5%) in CrossFit® strength training. The time between injury and surgical treatment was on average 10 months (3–60 months). Anabolic steroid use was reported by 87.5% of the surgical patients (35 cases).

**Table 3:** Characteristics and surgical outcomes of the patients.

	Total (n = 40)	Group		*P*-value
		Fascia lata (n = 20)	Semitendinosus/gracilis (n = 20)	
Age (years)				0.336^a^
Mean (SD)	32.9 (6.7)	33.9 (6.2)	31.9 (7.1)	
Minimum; maximum	22; 44	22; 42	44; 44	
Modality				0.407 ^b^
CrossFit	7 (17.5%)	2 (10.0%)	5 (25.0%)	
Weight training	33 (82.5%)	18 (90.0%)	15 (75.0%)	
History of steroid use				> 0.999 ^b^
No	5 (12.5%)	2 (10.0%)	3 (15.0%)	
Yes	35 (87.5%)	18 (90.0%)	17 (85.0%)	
BAK criteria				0.853 ^b^
E	28 (70.0%)	15 (75.0%)	13 (65.0%)	
F	2 (5.0%)	1 (5.0%)	1 (5.0%)	
G	10 (25.0%)	4 (20.0%)	6 (30.0%)	
Isokinetic test at 6 months				0.231 ^c^
Median (Q1; Q3)	8.0 (4.5; 10.0)	8.0 (3.0; 9.5)	8.5 (5.0; 12.0)	
Minimum; maximum	−19.0; 16.0	−19.0; 15.0	−10.0; 16.0	

Q1: first quartile; Q3: third quartile.

^a^Student’s *t*-test.

^b^Fisher’s exact test.

^c^Non-parametric Mann–Whitney test.

## Results

The mean age of the patients (n = 20) (Table 4) undergoing PMM reconstruction with a fascia lata graft [20] was 33.9 years, with an SD of 6.2. The time from injury to surgery ranged from 4 to 60 months, with a mean of 19.95 months and an SD of 14.3. The mean age of the patients (n = 20) (Table 5) undergoing PMM reconstruction with hamstrings [20] was 31.9 years. The time from injury to surgery ranged from 4 to 55 months. The time from injury to surgery ranged from 4 to 60 months, with a mean of 19.4 months and an SD of 7.1.

**Table 4:** Table showing type of fascia lata grafts, age, time from injury to surgery and location, sports modality, use of anabolic steroids, functional criteria and isokinetic evaluation.

Fascia lata	Age	Injury to surgery time (months)	Modality	History of steroid use	Bak criteria	Deficit isokinetic test of the shoulder at 6 months
1	41	12	Weight training	Y	G	12
2	32	6	Weight training	Y	E	8
3	37	24	Weight training	Y	G	10
4	25	13	Weight training	Y	E	9
5	33	9	Weight training	Y	E	0
6	37	36	Weight training	Y	R	18
7	23	32	Weight training	Y	E	4
8	22	18	Crossfit	Y	E	6
9	36	20	Weight training	Y	E	8
10	42	22	Weight training	N	G	10
11	34	8	Weight training	Y	F	15
12	38	10	Weight training	N	E	8
13	33	12	Weight training	Y	E	2
14	26	18	Crossfit	Y	E	0
15	30	9	Weight training	Y	R	19
16	39	4	Weight training	Y	E	5
17	41	38	Weight training	Y	R	18
18	34	60	Weight training	Y	E	5
19	33	38	Weight training	Y	G	10
20	42	10	Weight training	Y	E	8

**Table 5:** Showing type of hamstrings grafts, age, time from injury to surgery and location, sports modality, use of anabolic steroids, functional criteria and isokinetic evaluation.

SM/G	Age	Injury to surgery time (months)	Modality	History of steroid use	Bak criteria	Isokinetics deficit at 6 months
1	32	12	Weight training	Y	G	10
2	25	6	Weight training	Y	E	6
3	22	23	Weight training	Y	E	5
4	35	13	Weight training	Y	E	0
5	28	9	Crossfit	Y	E	−10
6	23	35	Weight training	Y	G	15
7	37	31	Weight training	Y	E	7
8	42	18	Crossfit	Y	E	8
9	38	20	Crossfit	Y	E	12
10	40	21	Weight training	N	G	16
11	24	8	Weight training	Y	E	4
12	28	10	Weight training	Y	G	12
13	22	12	Weight training	Y	E	1
14	41	17	Weight training	N	E	9
15	44	9	Crossfit	N	G	12
16	32	4	Crossfit	Y	F	15
17	37	38	Weight training	Y	E	5
18	32	55	Weight training	Y	E	5
19	30	37	Weight training	Y	G	11
20	25	10	Weight training	Y	E	10

**Figure 5:**
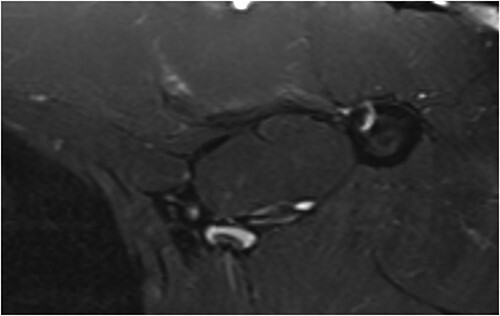
MRI of a patient 1 year post-operatively who underwent reconstruction surgery of the pectoralis major muscle tendon with fascia lata, showing a good image of tendon healing.

The Bak functional criteria [2] for patients receiving a fascia lata autograft showed 75% [15] of the patients presented excellent results (Fig. 5), 20% [4] presented good results, 5% [1] presented regular/fair results. In the hamstring group 65% [13] of the patients presented excellent results, 30% [6] presented good results and 5% [1] presented fair results (Table 4).

Isokinetic evaluation of these cases surgically treated at 6 months with a fascia lata graft revealed a peak torque at 60 degrees/s ranging from 0 to 19 N/s, with a mean of 8 N/s. In the hamstring group the mean was 8.5 N/s .

In the comparative analysis between groups, we did not find significant differences in patient characteristics and post-operative outcomes between the group that received fascia lata grafts and the group that received semitendinosus/gracilis grafts (Table 5), in relation to (*P* > 0.05 in all comparisons), which are described in detail in Tables 3–5.

Additionally, no significant differences were found between the operated and non-operated sides of the participants in the control group regarding the muscle degeneration measurements (Table 6) Figs 2–4 or the PMM MRI protocol, i.e. the largest PMM area (*P* = 0.251), PMM volume (*P* > 0.999), and humeral PMM insertion area (*P* = 0.136).

**Table 6:** Pathologic muscle degeneration findings in the muscle fragments of athletes with pectoralis major rupture.

Muscle Degeneration	N (%)
Atrophic fibres	
Absent	8 (53.3)
Mild (<25%)	7 (46.7)
Ischemic changes	
Absent	10 (66.7)
Present	5 (33.3)
Fatty Infiltration	
Absent	15 (100.0)
Other findings	
Sporadic hypotrophic fibres	1 (6.7)
Sporadic hypotrophic fibres + focal ischemic changes	3 (20.0)

The histological analysis of 15 muscle fragments (Table 6 and Fig. 2) showed We observed no atrophic fibres in eight patients (53%) and we observed moderate atrophic fibres in seven patients (46%) of the fragments, all of which were mild cases (< 25%). We observed ischemic changes in five (33.3%) fragments, and no fragments showed fatty infiltration (Fig. 3a and b). Other findings included four (26.7%) cases of sporadic hypertrophic fibres and three (20.0%) cases of focal ischemic changes.

## Discussion

The treatment of choice for total or almost total rupture of the PMM tendon is surgery, especially in athletes or those who practice physical activity and who require strength in the upper limbs [3–7, 15–18, 21–25].

In this study, PMM tendon reconstruction showed good results even when performed in chronic cases up to 1 year after injury. We obtained 60% excellent results according to Bak’s criteria [2] and 20% good results in cases that received fascia Lata grafts. In the hamstring graft group, 65% [13] of the patients presented excellent results, 30% [6] presented good results and 5% [1] presented fair results.

No statistically significant difference was found between the techniques using fascia lata grafts and knee flexors graft in several aspects (Table 3). However, we observed early weight-bearing and ambulation without significant pain in the fascia lata group (Figs 4 and 5), compared to the flexor group. In this group we found persistent pain lasting 2 weeks upon weight-bearing, even with seroma in four patients in the fascia lata graft group.

We believe that the presence of the clavicular portion in all of our cases, even if small, can maintain muscle activity and thus the viability of the injured PMM, preventing definitive degeneration [10]. Ejnisman and Pochini [10] showed similar electromyographic activity in the operative and contralateral sides in patients in the late post-operative period after PMM injuries [7].

The limitations of this study are surgical allocation per convenience, according to the availability of surgical material and, the small number of cases in this series, despite pectoralis rupture also does not represent such a common injury.

## Conclusion

Both surgical techniques studied using semitendinosus and fascia lata grafts obtained good results without statistical difference and proved to be a good option for reconstructing the tendon of the PMM.

Muscle samples and MRI obtained from the patients in this study showed no or few histological and image signs of muscle degeneration.

## References

[1] Angelo A , Azevedo C. Minimally invasive fascia lata harvesting in ASCR does not produce significant donor site morbidity. Knee Surg Sports Traumatol Arthrosc 2019;27:245–50. 30069653 10.1007/s00167-018-5085-1

[2] Bak K , Cameron EA, Henderson IJ. Rupture of the pectoralis major: a meta-analysis of 112 cases. Knee Surg Sports Traumatol Arthrosc 2000;8:113–9. 10795675 10.1007/s001670050197

[3] Connell DA , Potter HG, Sherman MF, Wickiewicz TL. Injuries of the pectoralis major muscle: evaluation with MR imaging. Radiology 1999;210:785–91. 10207482 10.1148/radiology.210.3.r99fe43785

[4] de Castro PA, Andreoli CV, Belangero PS, et al. Clinical considerations for the surgical treatment of pectoralis major muscle ruptures based on 60 cases: a prospective study and literature review. Am J Sports Med 2014;42:95–102. 24192390 10.1177/0363546513506556

[5] Schepsis AA , Grafe MW, Jones HP, Lemos MJ. Rupture of the pectoralis major muscle. Outcome after repair of acute and chronic injuries. Am J Sports Med 2000;28:9–15. 10653537 10.1177/03635465000280012701

[6] Sephien A , Orr J, Remaley DT. Pectoralis major tear in a 23-year-old woman while performing high-intensity interval training: a rare presentation. BMJ Case Rep 2020;13:e232649. 10.1136/bcr-2019-232649. PMC7101035 32193177

[7] de Castro PA, Ejnisman B, Andreoli CV, et al. Exact moment of tendon of pectoralis major muscle rupture captured on video. Br J Sports Med 2007;41:618–9. 17337486 10.1136/bjsm.2006.033563 PMC2465400

[8] de Castro PA, Ejnisman B, Andreoli CV, et al. Pectoralis major muscle rupture in athletes: a prospective study. Am J Sports Med 2010;38:92–8. 19880715 10.1177/0363546509347995

[9] Ejnisman E , Pochini AC, Andreoli CV. Electromyography of the pectoralis major muscle after surgical reconstruction of chronic tendon rupture. Rev Bras Ortop 2021;56:31–5. 10.1055/s-0040-1713387 PMC7895631 33627896

[10] Ejnisman B , Andreoli CV, Pochini AC, et al. Ruptura do musculo peitoral maior em atletas. Rev Bras Ortop. 2002;37:482–8.

[11] Pochini AC , Ferretti M, Kawakami EF, et al. Analisys of pectoralis major tendon in weightlifting athletes using ultrasonography and elastography. Einstein (Sao Paulo) 2015;13:541–6. 26761551 10.1590/S1679-45082015AO3335 PMC4878628

[12] Godoy IRB , Rodrigues TC, Skaf AY, et al. Bilateral pectoralis major MRI in weightlifters: findings of the non-injured side versus age-matched asymptomatic athletes. Skeletal Radiol 2022;51:1829–36. 10.1007/s00256-022-04031-7. 35303115

[13] Figueiredo EA , Terra BB, Cohen C, et al. Footprint do tendão do peitoral maior: estudo anatômico. Rev Bras Ortop 2013;48:519–23. 31304163 10.1016/j.rboe.2013.12.009 PMC6565948

[14] Goutallier D , Postel JM, Bernageau J, et al. Fatty muscle degeneration in cuff ruptures. Pre- and postoperative evaluation by CT scan. Clin Orthop 1994;304:78–83. 8020238

[15] Messer D , Shield A. Hamstring muscle activation and morphology are significantly altered 1-6 years after anterior cruciate ligament reconstruction with semitendinosus graft. Knee Surg Sports Traumatol Arthrosc 2020;28:733–41. 31030253 10.1007/s00167-019-05374-w

[16] Godoy IRB , Silva RP, Rodrigues TC, et al. Automatic MRI segmentation of pectoralis major muscle using deep learning. Sci Rep 2022;12:5300. 10.1038/s41598-022-09280-z. 35351924 PMC8964724

[17] Ohashi K , El-Khoury GY, Albright JP, Tearse DS. MRI of complete rupture of the pectoralis major muscle. Skeletal Radiol 1996;25:625–8. 8915045 10.1007/s002560050148

[18] Pochini AC , Rodrigues MSB, Yamashita L, et al. Surgical treatment of pectoralis major muscle rupture with adjustable cortical button. Rev Bras Ortop. 2018;53:60–6. 29367908 10.1016/j.rboe.2017.11.005 PMC5771794

[19] Rabuck SJ , Lynch JL, Guo X. Tendon rupture using hamstring autograft: a case report. Am J Sports Med 2006;34:295–8. 16170043 10.1177/0363546505278697

[20] Heikkinen J , Lantto I, Piilonen J, et al. Tendon length, calf muscle atrophy, and strength deficit after acute achilles tendon rupture: long-term follow-up of patients in a previous study. J Bone Joint Surg Am 2017;99:1509–15. 28926379 10.2106/JBJS.16.01491

[21] Uchiyama Y , Miyazaki S, Tamaki T, et al. Clinical results of a surgical technique using endobuttons for complete tendon tear of pectoralis major muscle: report of five cases. Sports Med Arthrosc Rehabil Ther Technol 2011;3:20. 21955511 10.1186/1758-2555-3-20 PMC3199274

[22] Tay VS-L , Loh ICY. Minimally invasive fascia Lata harvest: a new method. Plast Reconstr Surg Glob Open 2013;1:e7–8. 10.1097/GOX.0b013e31828c4406 PMC4174173 25289201

[23] Pochini A , Ejnisman B, Andreoli CM, Cohen M. Reconstruction of the pectoralis major tendon using autologous grafting and cortical button attachment: description of the technique. Tech Shoulder Elbow Surg 2012;13:77–80.

[24] Pochini AC , Andreoli CV, Ejnisman B, Maffulli N. Surgical repair of a rupture of the pectoralis major muscle. BMJ Case Rep 2015;2015:bcr2013202292. 10.1136/bcr-2013-202292 PMC4342657 25716033

[25] Sikka R , Neault M, Guanche C. Reconstruction of the pectoralis major tendon with fascia lata allograft. Orthopaedics 2005;28:1199–201. 10.3928/0147-7447-20051001-19 16237886

